# Data Driven Estimation of Imputation Error—A Strategy for Imputation with a Reject Option

**DOI:** 10.1371/journal.pone.0164464

**Published:** 2016-10-10

**Authors:** Nikolaj Bak, Lars K. Hansen

**Affiliations:** 1 Center for Neuropsychiatric Schizophrenia Research (CNSR) & Center for Clinical Intervention and Neuropsychiatric Schizophrenia Research (CINS), Psychiatric Center Glostrup, Copenhagen University Hospitals, Mental Health Services, Capital Region of Denmark, Glostrup, Denmark; 2 Cognitive Systems, DTU Compute, Dept. Applied Mathematics and Computer Science, Technical University of Denmark, Kongens Lyngby, Denmark; Soochow University, CHINA

## Abstract

Missing data is a common problem in many research fields and is a challenge that always needs careful considerations. One approach is to impute the missing values, i.e., replace missing values with estimates. When imputation is applied, it is typically applied to all records with missing values indiscriminately. We note that the effects of imputation can be strongly dependent on what is missing. To help make decisions about which records should be imputed, we propose to use a machine learning approach to estimate the imputation error for each case with missing data. The method is thought to be a practical approach to help users using imputation after the informed choice to impute the missing data has been made. To do this all patterns of missing values are simulated in all complete cases, enabling calculation of the “true error” in each of these new cases. The error is then estimated for each case with missing values by weighing the “true errors” by similarity. The method can also be used to test the performance of different imputation methods. A universal numerical threshold of acceptable error cannot be set since this will differ according to the data, research question, and analysis method. The effect of threshold can be estimated using the complete cases. The user can set an *a priori* relevant threshold for what is acceptable or use cross validation with the final analysis to choose the threshold. The choice can be presented along with argumentation for the choice rather than holding to conventions that might not be warranted in the specific dataset.

## Introduction

Missing data is an issue of significant interest in a broad range of research areas. The mechanism behind the missing data can have implications for subsequent analysis. Missing data problems are complex and typically divided into three categories: 1) dependent on the missing value itself, (referred to as “missing not at random”, MNAR), 2) dependent on observed values (“missing at random”, MAR), or 3) “missing completely at random” (MCAR). Numerous strategies have been developed for dealing with missing data; the maybe simplest approaches are to use only the ‘complete cases’, i.e., reducing the dataset to the cases with no missing data, use an indicator variable or exclude troublesome variables from the analysis. These methods can lead to biased results, especially, if the missing data are not MCAR (e.g. [1, 2]). Another way of dealing with missing data is to impute the missing data points. There are several methods for imputation (see [3] or [4] for reviews). In single imputation (e.g., mean imputation), a new complete dataset is generated by inserting an estimated value in place of each missing value. In multiple imputation (MI) [3] several datasets are generated, where the inserted values are drawn from the posterior predictive distribution of the missing variables from each case. These datasets are then used for further analysis. The bias is reduced in multiple imputation and more valid estimates of the mean and variance of sample summary statistics are obtained. In univariate analyses based on limited samples it seems that imputation is preferable to complete case analysis (e.g. [5, 6]). In these cases the goal of the imputation is not to determine the exact value of each missing value, but rather to estimate them so that the characteristics of the variable are conserved. The analyses can then be performed on the whole dataset without being biased by the missing values. However, when multivariate multimodal datasets are investigated, e.g., in machine learning approaches, the actual values in each observation can be very important and the literature is sparse. During imputation several questions appear: Is too much missing? Is the number of missing variables important or are there specific variables that we should worry about when missing? Will the use of imputation change subsequent analysis, i.e., lead to changed classification? The answers to these questions will depend on the selected imputation technique and the data [7]. One approach suggested by Clavel and colleagues to handle the above mentioned questions is to use the uncertainty of the multiple imputation technique to estimate the confidence intervals of each imputed value, along with the effect it has on the overall analysis. Then observations with (too) large confidence interval can be removed iteratively taking into account possible transformations of the data (e.g. mean and variance) when changing the dataset [8]. In this work we also aim to identify uncertain imputations to provide the user with a practical tool for evaluating imputations. It addresses the question of how many or which features can be missing and an aggregated score can still be imputed. It can also be used to evaluate different imputation algorithms on a dataset. We do this by simulating missing values in the complete cases, the estimated error of imputation for each observation with missing values can then be calculated. The approach is related to the “Full Mechanism Bootstrap” method for variance estimation [9, 10], in that the patterns of missing values in the data, the ‘missingness patterns’ (e.g. the missingness pattern for case (*x*_1_, *x*_2_, −, *x*_4_, *x*_5_, *x*_6_, −) would be (1, 1, 0, 1, 1, 1, 0)) are simulated. Our method, however focuses on the error and variance of the actual imputation algorithm rather than estimating the variance of the variables with missing values. With this method it is still necessary to set a threshold or what is an acceptable error. The appropriate threshold depends on the data, the imputation method, and the subsequent analysis method. Based on variance of the complete cases the user could estimate an appropriate threshold for the acceptable error. This could be done *a priori*, before the analysis. Alternatively, the threshold could be set based on the analysis outcome e.g. subsequent classification error. Cases with missing that have an estimated error larger than the threshold are then excluded from the analysis. Using this strategy, the user can also report the specific threshold and method. This leads to a tool which we refer to as “imputation with reject option”. The method can be used to evaluate how accurately imputation algorithms perform in a specific dataset. Additionally, missingness patterns in the data that are too problematic to impute at all can be identified from the data rather than using a convention. The formal statistical considerations about missingness mechanism and whether to impute at all are still necessary. It is intended as an additional tool and not a replacement for the currently used techniques. It will be most appropriate in machine learning approaches in cases where MI is not appropriate e.g. classification paradigms where a one data point for each observation is necessary.

This method is but one of many methods developed to tackle issues in the data using machine learning methods. This is ongoing to improve algorithms and approaches in this field. Other issues in data that can compromise results include features that disappear or new features appearing in data coming as a stream (e.g. [11]), corrupted features that change in importance in a data stream (e.g. [12] Dekel, Shamir and Xiao, 2009), or missing labels, termed semi-supervised learning (e.g. [13]).

To illustrate the strategy, we use the following datasets: For comparison with previous work we analyse the dataset of Brown and collegues [7] used in [8]. In addition, we use an Echocardiogram dataset and the Chronic Kidney Disease dataset from the UCI machine learning repository [14, 15].

## Methods

Imputation with reject option is intended to be used to test a chosen imputation algorithm, or evaluate which imputation algorithm to use for imputation in a dataset. The methods does not replace the usual considerations about missingness mechanism and whether the complete cases are representative. Whether the data are MNAR should therefore be considered *before* implementation of the method. The method is based on the following assumptions:

- The missingness pattern is predictive of the accuracy i.e. cases with few features missing are more likely to be imputed with low error than cases with many missing features.
- If a specific missingness pattern is introduced in the complete cases, the resulting accuracy of imputation is predictive for the accuracy of imputation of cases with similar missingness patterns.
- Cases with similar feature values will be imputed with similar accuracies.

The method is thought to be generalizable and can be used with any imputation method but requires that the number of complete cases is high enough to be representative. With too few complete cases the estimated error will not be comparable to the actual difference between the true and imputerd data point, the ‘imputation error’. This should be tested with a learning curve comparing root mean square error (RMSE). For demonstration we use three single imputation techniques: 1. a probabilistic PCA model (PPCA) [16, 17], see S1 Appendix for details, 2. Mean imputation, where the mean of the feature is inserted in spaces with missing values, and 3. K-Nearest Neighbour imputation, where the mean of the 3 nearest neighbours’ values are inserted in spaces with missing values. The nearest neighbours were determined with euclidean distance based on the non-missing features. In all three imputation methods the 2D principal component space were determined by the complete cases.

Consider a N by D data matrix $\bar{\bar{X}}$ consisting of *N*_com_ complete cases, *N*_miss_ cases with M different missingness patterns represented as D dimensional vectors with zeros and ones marking missing or presence in each feature (${\bar{\mu }}_{1},{\bar{\mu }}_{2},\cdots ,{\bar{\mu }}_{M}$). Each complete case data point is used to simulate all missingness patterns present in the data. Each of these *N*_com_ ⋅ *M* “new” data points are then imputed, in a leave-one-out (LOO) [18] approach based on the other (*N*_com_ − 1) complete cases. At this point we then have *N*_com_ ⋅ *M* data points consisting of simulated missing data in the complete cases. These are then compared to the true value of the *N*_com_ complete cases to determine the errors for all missingness patterns for each complete case. An *N*_com_ ⋅ *M* matrix Err of errors are then determined. We now have an estimate of how accurate we can impute the complete cases if they had missing values. For each of the *N*_miss_ cases with missing, represented as the vector $\bar{x}$ the errror is estimated using its own data ($\bar{x}$), its missingness pattern (${\bar{\mu }}_{\bar{x}}$) and the *N*_com_ ⋅ *M* “true errors”. In short, the error for each case is estimated as a weighted average of the errors from the complete cases with simulated missing values. These errors are weighed based on similarity with the missingness patterns (so that the actual missingness pattern weighs most but similar patterns also have high weights) and the similarity of the complete cases (so complete cases that have similar values in the features present is weighed highest),

$$\begin{array}{c} \text{Estimated}{\text{Err}}_{\bar{x}}=\sum_{m=1}^{M}\frac{S_{1}\left({\bar{\mu }}_{\bar{x}},{\bar{\mu }}_{m}\right)}{\sum_{m^{\prime }=1}^{M}S_{1}\left({\bar{\mu }}_{\bar{x}},{\bar{\mu }}_{m^{\prime }}\right)}\sum_{n=1}^{N_{\text{com}}}\frac{S_{2}\left(\bar{x},{\bar{x}}_{n}\right)}{\sum_{n^{\prime }=1}^{N_{\text{com}}}S_{2}\left(\bar{x},{\bar{x}}_{n^{\prime }}\right)}{\text{Err}}_{n,m}. \end{array}$$ (1)

Where Err_*n*,*m*_ is the error of complete case n with the missingness pattern m. The vector ${\bar{\mu }}_{m}$ is the *m*’th missingness pattern type, while ${\bar{x}}_{n}$ is the *n*’th complete observation vector, and *S*_1_ and *S*_2_ are the similarity measures for missingness pattern and feature values respectively. As error measure we used the Euclidean distance between imputed and true data points in the 2D PCA space.

$$\begin{array}{c} S_{1}\left({\bar{\mu }}_{\bar{x}},{\bar{\mu }}_{m}\right)=e^{-\gamma \left|\right|{\bar{\mu }}_{\bar{x}}-{\bar{\mu }}_{m}\left|\right|}, \end{array}$$ (2)

and

$$\begin{array}{c} S_{2}\left(\bar{x},{\bar{x}}_{n}\right)=e^{-\delta \left|\right|{\bar{\mu }}_{\bar{x}}⊙\left(\bar{x}-{\bar{x}}_{n}\right)\left|\right|}, \end{array}$$ (3)

where $\left|\right|{\bar{\mu }}_{\bar{x}}⊙\left(\bar{x}-{\bar{x}}_{n}\right))||$ calculates the Euclidean distance only from present features, and *γ* and *δ* are scale parameters. The scale parameters adjusts the similarity measure, higher values gives more weight to the most similar values. In our examples we performed a simple grid search to optimise these variables testing *γ* ∈ [0, 10] and *δ* ∈ [0, 10].

The effects of the threshold can be explored using the complete cases with simulated missing values. By replacing *N*_com_ in Eq (1) the same approach can be used to calculate the estimated error for cases with simulated missing values. The effect of threshold can then be visualized by plotting the threshold against both the RMSE of the cases that would be included and those that would be excluded at the given threshold. Additionally, it can be visualized how well the estimated error compares to the actual imputation error. Setting a threshold to what is acceptable is still determined by the user although with this method it is set for the estimated error based on the data rather than a general rule of thumb. The threshold can be based on the the estimated error or also include the variation of the estimated errors. It can be based on the absolute value or it could be normalized (e.g. Estimated Err < 0.5 or Estimated Err < 1%). As mentioned above, the method require a representative number of complete cases. With to few cases the imputation will be unreliable. This is always true when using imputation, but with this method the number of complete cases is critical since these are used for both estimating the error, setting the threshold, and imputing the cases with missing values. It is therefore important to test the effect of the number of complete cases. This can be done by creating a learning curve with a variable number of the complete cases included. We used the RMSE of the estimated errors and the actual imputation error of the complete cases in our learning curve. The learning curve was created with 100 replications. Both the RMSE of the estimated and the actual errors should be very close to their asymptotic values to indicate that additional complete cases would not improve the model, and a sufficient number of complete cases are present.

A user can then report as a minimum that this method was used, which similarity measures were used, the number of included and excluded cases with missing values, the chosen threshold of estimated error, and the RMSE at this threshold. This will assist scientific reproducibility.

### Data

#### Crocs dataset

The dataset of Brown et al. [7] consists of information from crocodilian crania, from 226 specimens with 23 cranial measurements as features. The dataset was retrieved from: http://dx.doi.org/10.5061/dryad.m01st7p0. This dataset does not contain missing data, so this was simulated twice using the “Obliterator” and “Byclade” tools. Both functions from the “LOST” package freely available on line from the CRAN (Comprehensive R Archive Network; http://www.rproject.org. Both tools create datasets with MNAR values. Obliterator creates missing values with anatomical bias, simulating missing parts of the crocodile crania, while Byclade creates missing values with species bias, with higher probability of missing values in rare species. The fraction of missing values was set to 50% in the complete dataset for both mechanisms. The functions created datasets with 50% missing values but with no complete cases. The proposed strategy in this work requires a substantial set of complete cases so datasets consisting of 40% (randomly chosen) missing data observations and 60% complete case observations were used for the analyses (see S1 Data for the pattern of missing in the complete dataset).

#### Echocardiogram dataset

The second dataset was retrieved from the Machine Learning Database Repository at the University of California, Irvine [14]. The echocardiogram dataset. The dataset contains 132 observations, the first nine features were used. Missing data were already present in the data, so no simulation of missing was performed, the dataset contained 26 observations with missing values.

#### Chronic kidney disease dataset

The third dataset was retrieved from the Machine Learning Database Repository at the University of California, Irvine [14]. Chronic kidney disease dataset. The dataset contains 400 observations, all eleven numeric features were used. Missing data were already present in the data, so no simulation of missing was performed, the dataset contained 185 observations with missing values.

## Results

### Crocs dataset

**Fig 1:**
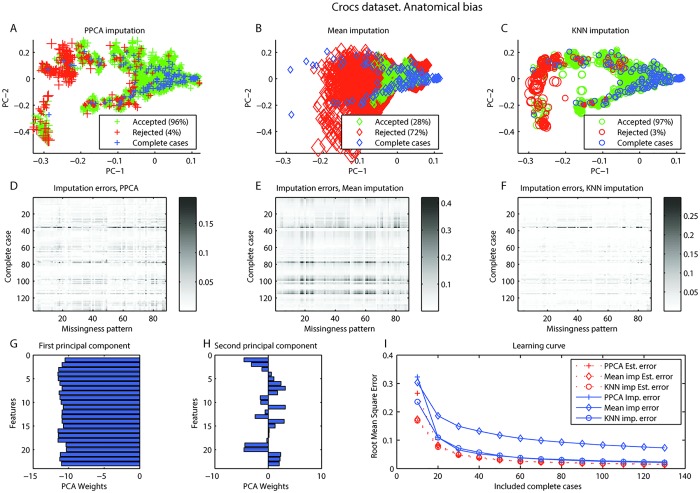
Crocs dataset, Anatomical bias. Crocs dataset, Anatomical bias. A-C. Visualization of imputation of complete cases with simulated missing values for A. Probabilistic PCA (PPCA) imputation, B. Mean imputation, C. KNN imputation. In all three, blue represent complete cases. Simulated cases with missing that would be rejected (red) or accepted (green) for estimated error <0.03. The size represents the actual imputation error. D-F. Shows the imputation errors for all complete cases (rows) with all missingness patterns simulated (columns) for D. PPCA imputation, E. Mean imputation, F. KNN imputation. G-H. Feature weights in first and second principal components. I. Learning curve presenting root mean square error (RMSE) as a function of included cases with 100 replications at each step. RMSE was calculated both the estimated errors and the actual imputation errors.

**Fig 2:**
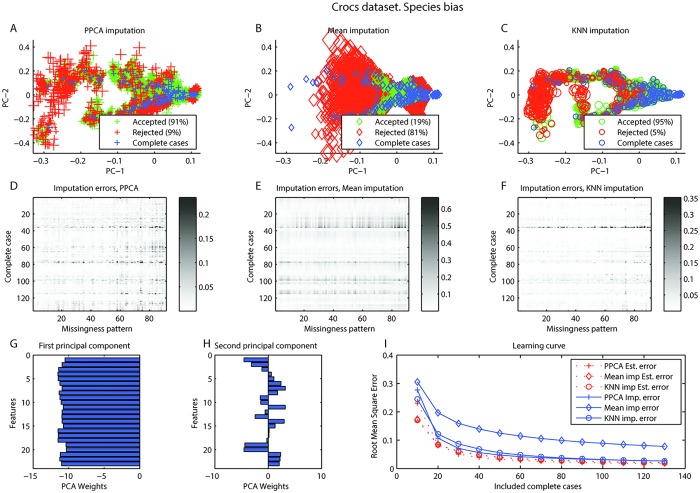
Crocs dataset, Species bias. Crocs dataset, Species bias. A-C. Visualization of imputation of complete cases with simulated missing values for A. Probabilistic PCA (PPCA) imputation, B. Mean imputation, C. KNN imputation. In all three, blue represent complete cases. Simulated cases with missing that would be rejected (red) or accepted (green) for estimated error <0.03. The size represents the actual imputation error. D-F. Shows the imputation errors for all complete cases (rows) with all missingness patterns simulated (columns) for D. PPCA imputation, E. Mean imputation, F. KNN imputation. G-H. Feature weights in first and second principal components. I. Learning curve presenting root mean square error (RMSE) as a function of included cases with 100 replications at each step. RMSE was calculated both the estimated errors and the actual imputation errors.

**Fig 3:**
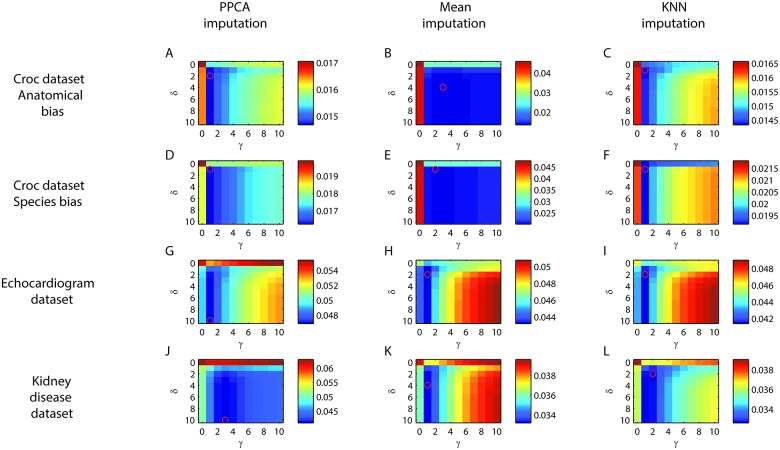
Gridsearch. Gridsearch to estimate *γ* and *δ* for each dataset. Optimal parameters indicated with a red circle. A-C. Crocs dataset with anatomical bias imputed with PPCA (A), Mean imputation (B), or KNN imputation (C). D-E Crocs dataset with species bias imputed with PPCA (D), Mean imputation (E), or KNN imputation (F). G-I Echocardiogram dataset imputed with PPCA (G), Mean imputation (H), or KNN imputation (I). J-L Chronic kidney disease dataset imputed with PPCA (J), Mean imputation (K), or KNN imputation (L).

The first principal component loaded on all features, possibly representing the size of the specimen, whereas the second principal component was distributed unevenly, representing shape of the specimen (Figs 1G and 1H and 2G and 2H). Optimal values for *γ* and *δ* in both anatomical and species bias are were found for all imputation types with a grid search (Fig 3A–3F).

For anatomical bias in the croc dataset, it can be seen that PPCA and KNN performs better than mean imputation and would reject fewer of the cases with simulated missing (Fig 1A–1C). It is also clear that certain missingness patterns (vertical lines) and certain complete cases (horizontal lines) could be problematic with in all imputation methods (Fig 1D–1F). The learning curves are close to the apparent asymptotic value so the sample seems to have sufficient size for the model (Fig 1I). The results are similar for species bias set. Again, mean imputation performs poorly, but here KNN seems to perform better than PPCA (Fig 2A–2C). The species bias dataset seems more difficult to impute than the anatomical bias dataset. Certain missingness patterns and certain complete cases are also in this case problematic to impute regardless of imputation type (Fig 2C–2F). However, this is not likely due to a small sample as the learning curve seems to have reached a stable level (Fig 2I). The effect of different thresholds in the croc dataset are presented in S1 and S2 Videos. A user might choose to accept only observations with low estimated error e.g. accepting only observations with Estimated Err < 0.03. With value of the threshold 90, 20 or 85 of 91 would be accepted with PPCA, mean imputation or KNN imputation respectively for anatomical bias. For species bias the number would be 85, 14 or 87 of 91. The choice of threshold would be dependent on the subsequent analysis.

### Echocardiogram dataset

**Fig 4:**
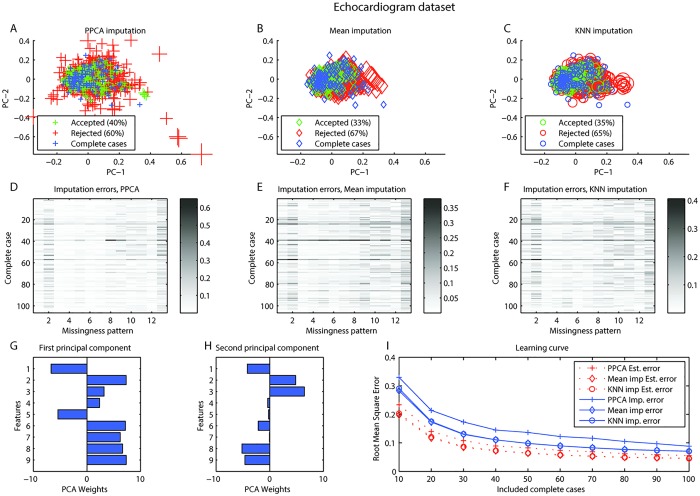
Echocardiogram dataset. Echocardiogram dataset. A-C. Visualization of imputation of complete cases with simulated missing values for A. Probabilistic PCA (PPCA) imputation, B. Mean imputation, C. KNN imputation. In all three, blue represent complete cases. Simulated cases with missing that would be rejected (red) or accepted (green) for estimated error <0.03. The size represents the actual imputation error. D-F. Shows the imputation errors for all complete cases (rows) with all missingness patterns simulated (columns) for D. PPCA imputation, E. Mean imputation, F. KNN imputation. G-H. Feature weights in first and second principal components. I. Learning curve presenting root mean square error (RMSE) as a function of included cases with 100 replications at each step. RMSE was calculated both the estimated errors and the actual imputation errors.

The first principal component corresponds to basic relations between survival and the physiological measures (Fig 4G and 4H). With the gridsearch the optimal values for *γ* and *δ* were found (Fig 3G–3I). In the echocardiogram dataset it can be seen clearly that certain patterns of missingness have higher error and cannot be imputed with the PPCA model or mean imputation. Also in this dataset certain complete cases seem problematic (Fig 4D–4F). Setting the same threshold as in the crocs dataset (Estimated Err < 0.03) would result in imputing only 11 of 26 cases for PPCA, 8 for mean imputation, and 10 would be imputed with KNN imputation (see S3 Video for visualization of the effect of threshold). The learning curves reach the asymptotic values indicating that increased sample size would not improve the model (Fig 4I).

### Chronic kidney disease dataset

**Fig 5:**
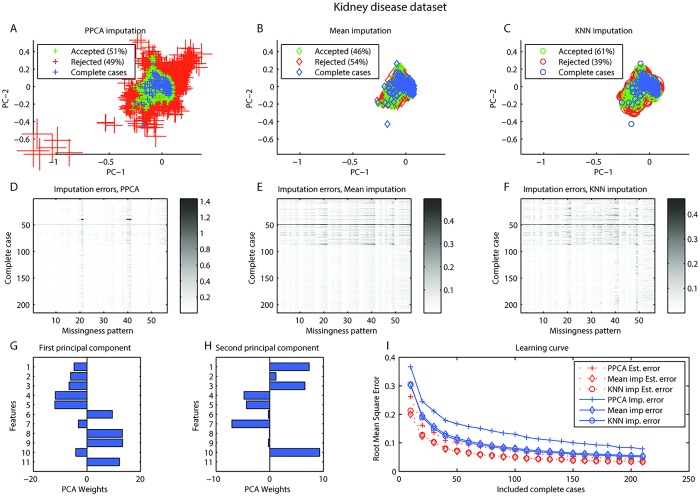
Chronic kidney disease dataset. Chronic kidney disease dataset dataset. A-C. Visualization of imputation of complete cases with simulated missing values for A. Probabilistic PCA (PPCA) imputation, B. Mean imputation, C. KNN imputation. In all three, blue represent complete cases. Simulated cases with missing that would be rejected (red) or accepted (green) for estimated error <0.03. The size represents the actual imputation error. D-F. Shows the imputation errors for all complete cases (rows) with all missingness patterns simulated (columns) for D. PPCA imputation, E. Mean imputation, F. KNN imputation. G-H. Feature weights in first and second principal components. I. Learning curve presenting root mean square error (RMSE) as a function of included cases with 100 replications at each step. RMSE was calculated both the estimated errors and the actual imputation errors.

The first principal component corresponds to links between lower Age, Blood pressure, Blood glucose, Blood urea, and Serum creatinine with higher Sodium, Hemoglobin, Packed cell volume, and Red blood cell count. The second principal component explains variance associated with higher Age, Blood glucose and White blood cell count, and lower Blood urea, Serum creatinine and Potassium (Fig 5G and 5H). With the gridsearch the optimal values for *γ* and *δ* were found (Fig 3J–3L). In the Chronic kidney disease dataset dataset it can be seen clearly that one case cannot be imputed correctly, regardless of which missingness pattern are simulated or which imputation algorithm are used (horizontal line). Certain patterns of missingness also have higher error and cannot be imputed with the PPCA model, mean, or KNN imputation (Fig 5D–5F). Setting the threshold as before (Estimated Err < 0.03) would result in imputing only 101 of 185 cases for PPCA, and 86 and 105 would be imputed with mean and KNN imputation (see S4 Video for visualization of the effect of threshold). The learning curve does not reach the asymptotic value for PPCA indicating that the number of complete cases might be too small and an increase in sample size could improve the model (Fig 5I). PPCA do not impute this dataset accurately when two dimensions are used (Fig 5A).

## Discussion

The missing data problem is addressed in many fields. Previous work has focused on judging the reliability of imputation based on the proportion of missing data in the dataset (e.g. [7, 19]). Such general guidelines could be problematic as the error of imputation will depend on the data and the imputation method. Clavel et al. advanced the state of the art by evaluating the uncertainty in imputation due to proportion of missing and missingness patterns in a dataset. They investigated different MI methods, the number of multiple imputations, and used the uncertainty to assess the individuals reliability of imputation [8]. We used three datasets: for comparison with Clavel et al., the “crocodile” dataset, and additionally the echocardiogram dataset [14] and the chroonic kidney disease dataset [14]. Imputation with a reject option is a generalized method, what is referred to as a “wrapper” function in machine learning, in that it can be used for any single imputation method. We tested three single imputation methods, PPCA, mean imputation, and KNN imputation. The results support our three assumptions. The vertical patterns in panels D, E, and F in in Figs 1, 2, 4, and 5 reveals that some missingness patterns are imputed with low error, while others have higher errors regardless which complete case are used to simulate the pattern. Similarly, horizontal patterns in these panels reveal that some complete cases are imputed with higher error while others are imputed with lower error regardless of the missingness pattern simulated.

In the presented examples the imputation was done into the principal component space based on complete cases. This way additional complexity by Procrustes rotation of the PCA space (i.e. that the PCA space changes if the PCA is performed on a subsample) is avoided since the imputed cases are not used for estimating the PCA. If the cases with missing values are used in the PCA, Procrustes rotation have to be considered and solved with e.g. with Procrustes superimposition with multiple imputation [8, 20, 21]. The new method is thought to be a generalizable strategy that can be used with any imputation method and it could be used in many fields. If the cross validated learning curve indicates that there are too few complete cases simulated data could be used instead of the original complete cases, however, this was not tested in the present paper.

Another use of the method is to test which imputation algorithm performs best in a given dataset. The strategy works for any imputation method since the algorithm itself is used to estimate the errors. For univariate analyses in a MI setting, this could be run first. The actual multiple imputation could then be performed with the most appropriate algorithm for the given dataset.

The error measure should be chosen to reflect the given application. The effect of threshold on the complete cases with simulated missing should be investigated when setting a threshold. A threshold for what is acceptable can then be set based on either the knowledge of the imputed variable by visualization, or it could be based on quantive estimation by cross-validation on the subsequent analysis, e.g. classification. The user can report and argue exactly why this threshold was chosen. In some datasets it will be appropriate to optimize the threshold and the normalization parameters via cross-validation using downstream analysis such as classification to optimise them. In the present example we estimated the parameters with a gridsearch.

If new observations with missing data are acquired the same procedure can be used: the missingness pattern in the new observations is replicated in each complete case observation. Based on the calculated errors, the missingness pattern, similarity with complete cases, and the previously chosen threshold, the new observations can then be imputed or rejected.

Our strategy enables users to evaluate the imputations in their dataset. Rather than using a generic “rule of thumb”, a user can test and report how successful the chosen imputation method is on the specific dataset. A future development could be to calculate the probability of errors larger than a specific value for each missing case making it even easier to set and report a threshold.

## Supporting Information

S1 Appendix Description of the methods used for imputation with Probabilistic PCA. (PDF)

S1 Data Data zip-file that includes the missingness patterns for both anatomical bias and species bias creates with “Obliterator” and “Byclade” tools. The fraction of missing values was set to 50% in the complete dataset for both mechanisms. Then the final datasets were made consisting of 40% (randomly chosen) missing data observations and 60% complete case observations. The zip file includes two.txt files consisting of zeros and ones (226*23), where ones represents missing values. These can be overlain with the original data from http://dx.doi.org/10.5061/dryad.m01st7p0.
(ZIP)

S1 Video Effect of Threshold, Crocs dataset, Anatomical bias. Movie showing the effect of different thresholds (see Fig 1). A-C. Visualization of the effect of threshold using the complete cases with simulated missing values for A. Probabilistic PCA (PPCA) imputation, B. Mean imputation, C. KNN imputation. In all three, blue represent complete cases. Simulated cases with missing that would be rejected (red) or accepted (green) for the current threshold. The size represents the actual imputation error. D. Threshold versus an errors examplified with Root Mean Square Errors (RMSE) of the actual imputation error for the included cases (full red lines) and the excludedcases (dotted blue lines).
(AVI)

S2 Video Effect of Threshold, Crocs dataset, Species bias. Movie showing the effect of different thresholds (see Fig 2). A-C. Visualization of the effect of threshold using the complete cases with simulated missing values for A. Probabilistic PCA (PPCA) imputation, B. Mean imputation, C. KNN imputation. In all three, blue represent complete cases. Simulated cases with missing that would be rejected (red) or accepted (green) for the current threshold. The size represents the actual imputation error. D. Threshold versus an errors examplified with Root Mean Square Errors (RMSE) of the actual imputation error for the included cases (full red lines) and the excludedcases (dotted blue lines).
(AVI)

S3 Video Effect of Threshold, Echocardiogram dataset. Movie showing the effect of different thresholds (see Fig 4). A-C. Visualization of the effect of threshold using the complete cases with simulated missing values for A. Probabilistic PCA (PPCA) imputation, B. Mean imputation, C. KNN imputation. In all three, blue represent complete cases. Simulated cases with missing that would be rejected (red) or accepted (green) for the current threshold. The size represents the actual imputation error. D. Threshold versus an errors examplified with Root Mean Square Errors (RMSE) of the actual imputation error for the included cases (full red lines) and the excludedcases (dotted blue lines).
(AVI)

S4 Video Effect of Threshold, Kidney disease dataset. Movie showing the effect of different thresholds (see Fig 5). A-C. Visualization of the effect of threshold using the complete cases with simulated missing values for A. Probabilistic PCA (PPCA) imputation, B. Mean imputation, C. KNN imputation. In all three, blue represent complete cases. Simulated cases with missing that would be rejected (red) or accepted (green) for the current threshold. The size represents the actual imputation error. D. Threshold versus an errors examplified with Root Mean Square Errors (RMSE) of the actual imputation error for the included cases (full red lines) and the excludedcases (dotted blue lines).
(AVI)
