# Supplementary material for: Data Driven Estimation of Imputation Error—A Strategy for Imputation with a Reject Option
Source: PLoS One. 2016 Oct 10;11(10):e0164464. doi: 10.1371/journal.pone.0164464 (PMC5056679; doi:10.1371/journal.pone.0164464)
Supplement: S1 Appendix — (PDF) [file pone.0164464.s001.pdf]

Supporting information for Data driven estimation of imputation error -A strategy for imputation with a reject option

S1 Appendix

Imputation with Probabilistic PCA

We focus on latent variable models for dimensional reduction; in case so-called "probabilistic principal component analysis" (for a general discussion and references to original work see [2]). Under the probabilistic PCA model data are generated by the process

$$\bar{x} = \bar{\bar{A}}\bar{z} + \bar{\epsilon} \tag{1}$$

where the latent variables (principal components) are multivariate normal with unit covariance matrix $\bar{z} \sim \mathcal{N}(\bar{0}, I)$, and the additive noise is normal with variance σ^2, hence,$\bar{\epsilon} \sim \mathcal{N}(\bar{0}, \sigma^2 I)$. The observations are multivariate normal with covariance matrix

$$\bar{\bar{\Sigma}} = \bar{\bar{A}}\bar{\bar{A}}^T + \sigma^2 I. \tag{2}$$

Using

$$p\left(\bar{x}, \bar{z}|\bar{\bar{A}}, \sigma_0^2\right) = p\left(\bar{x}|\bar{z}\right) p\left(\bar{z}\right) \propto e^{-\frac{1}{2\sigma^2}||\bar{\bar{A}}\bar{z}-\bar{x}||^2} e^{-\frac{1}{2}\bar{z}^2}, \tag{3}$$

we get the distribution of the principal components conditioned on observations

$$\log p\left(\bar{z}|\bar{x}\right) = -\frac{1}{2\sigma^2}||\bar{\bar{A}}\bar{z} - \bar{x}||^2 - \frac{1}{2}\bar{z}^2 + \text{const.}, \tag{4}$$

$$= -\frac{1}{2\sigma^2}\bar{z}^T\bar{\bar{A}}\bar{\bar{A}}^T\bar{z} + \frac{1}{\sigma^2}\bar{x}^T\bar{\bar{A}}\bar{z} - \frac{1}{2}\bar{z}^2 + \text{const.}. \tag{5}$$

Hence, the conditional distribution of the principal components is the normal distribution $\mathcal{N}\left(\bar{\mu}_{z|x}, \bar{\bar{\Sigma}}_{z|x}\right)$, with

$$\bar{\mu}_{z|x} = \frac{1}{\sigma^2}\bar{\bar{\Sigma}}_{z|x}\bar{\bar{A}}^T\bar{x},$$
$$\bar{\bar{\Sigma}}_{z|x}^{-1} = \frac{\bar{\bar{A}}\bar{\bar{A}}^T}{\sigma^2} + I \tag{6}$$

Inference based on a sample of N complete data points $\bar{x} \in \mathbb{R}^d$ forming the data matrix

$$\bar{\bar{X}} = [\bar{x}_1, \bar{x}_2, \ldots, \bar{x}_N](centered) \tag{7}$$

is based on the singular value decomposition,

$$\bar{\bar{X}} = \bar{\bar{U}}\bar{\bar{S}}\bar{\bar{V}}^T. \tag{8}$$

Let k be the selected subspace dimension, then the subspace of interest is spanned by the columns of the matrix $\bar{\bar{A}}$ estimated by

$$\hat{\bar{\bar{A}}} = \bar{\bar{U}}_{1:k}\bar{\bar{S}}_{1:k}. \tag{9}$$

The noise variance is estimated from the variance outside the subspace of interest

$$\widehat{\sigma^2} = \frac{\mathrm{Tr}\,(\bar{\bar{X}}\bar{\bar{X}}^T) - \mathrm{Tr}\,(\bar{\bar{X}}_r\bar{\bar{X}}_r^T)}{N(d-k)}. \tag{10}$$

where the subspace reconstruction of data is given by

$$\bar{\bar{X}}_r = \bar{\bar{U}}_{1:k}\bar{\bar{U}}_{1:k}^T \cdot \bar{x}. \tag{11}$$

Now consider inference based on missing data, i.e., the remaining features indexed by the set m $\bar{x} \longrightarrow \bar{x}_m$. The relevant distribution of principal components conditioned on the features present $\bar{x}_m$ is given simply by $\mathcal{N}\left(\bar{\mu}_{z|\bar{x}_m}, \bar{\bar{\Sigma}}_{z|\bar{x}_m}\right)$, with

$$\widehat{\bar{\bar{\Sigma}}_{z|\bar{x}_m}^{-1}} = \frac{\hat{\bar{\bar{A}}}_m\hat{\bar{\bar{A}}}_m^T}{\widehat{\sigma^2}} + I \tag{12}$$

$$\widehat{\bar{\mu}_{z|\bar{x}_m}} = \frac{1}{\widehat{\sigma^2}}\widehat{\bar{\bar{\Sigma}}_{\bar{z}|\bar{x}_m}}\hat{\bar{\bar{A}}}_m^T\bar{x}_m \tag{13}$$

where $\hat{\bar{\bar{A}}}_m$ are the rows of subspace spanning vectors corresponding to the features present $\bar{x}_m$.

References

1. Bishop CM. Pattern Recognition and Machine Learning (Information Science and Statistics). Secaucus, NJ, USA: Springer-Verlag New York, Inc.; 2006.
